# Cognition and Vascular Risk Factors: An Epidemiological Study

**DOI:** 10.1155/2012/783696

**Published:** 2012-09-04

**Authors:** Augusto Vicario, Mildren Del Sueldo, Ruth A. Fernández, Julio Enders, Judith Zilberman, Gustavo H. Cerezo

**Affiliations:** ^1^Department of Internal Medicine, Cardiovascular Division, Hospital Español de Buenos Aires, Argentina; ^2^Argentine Federation of Cardiology (AFC), Buenos Aires, Argentina; ^3^Research Group, Human Health Commission, CERTUS Foundation, Villa María, Córdoba, Argentina; ^4^School Medicine, National University of Córdoba, Córdoba, Argentina; ^5^School of Public Health, School Medicine, National University of Córdoba, Córdoba, Argentina

## Abstract

We conducted an epidemiological approach to identify the negative impact of the vascular risk factors (such as hypertension, diabetes and hypercholesterolemia) over cognition. The interesting aspect of this study was that the survey was conducted in all age groups through a voluntary call (*n* = 1365; ≥18 years old, both sexes; age 49 ± 15 y, female 75.7%). Thus, we demonstrated that the use of a Minimum Cognitive Examination (MCE), a brief, simple, and easy managed neuropsychological evaluation, detected a greater number of people with cognitive decline surpassing to the Minimal Mental Statement Examination alone (14.5% of the participants showed MMSE ≤24, 34,6% showed dys-executive function, and 45,8% memory impairment. Out of the 4 studied RF, the only one that was not related to cognitive impairment was dyslipemia. Finally, we noted the importance of cognitive state early detection in all age groups, even in the youngest group. Acting in the middle of the life stages, we can prevent or delay the onset of a disease in adults, nowadays incurable: dementia.

## 1. Introduction

Alzheimer disease international (ADI) have issued three reports since 2009, creating awareness about the high prevalence and incidence of the Alzheimer Disease (AD), especially in the low-income countries (≥5% in Latin America on people over 65 years old), about the increase in the cost of dementia and about the importance of an early identification. Moreover, these reports mention the possibility of applying new preventing strategies, like exercise promotion and vascular risk factors (RFs) control [1]. Alzheimer's type dementia and vascular dementia share the same RF and there is anatomopathological evidence related to atherosclerosis [2, 3]. Likewise, vascular disorders seem to contribute to the risk of AD as well as its stage and diagnosis. If we accept that 20% of the patients who were diagnosed with mild cognitive impairment may evolve towards both conditions, the cognitive disorders early identification, especially in cohorts with vascular RF and/or diseases would be extremely important.

The relationship between age and cognitive performance is well known. Even though it is debatable, according to recent investigation the beginning of such cognitive decline may be fixed in middle-aged people [4]. Therefore, age, dementia, and RF may build a situation in which general practitioners and cardiologists cooperate in a multidisciplinary team (neurologists, psychiatrist, and neuropsychologists) where the main priority is “identifying”, “delaying”, and even “preventing” cognitive disorders.

Based on this background, the aim of our study is to identify cognitive disorders, as well as their relationship with the RF and verify the utility of the Minimum Cognitive Examination (MCE) as a tool in inhabitants from Villa María, Córdoba, Argentina.

## 2. Methods

### 2.1. Study Populations

The screening was done from February 2010 to August 2011. This study was a part of the Program “Corazón Sano” (Healthy Heart) a Cardiovascular Prevention Program set up by the Health Council of Villa María, Córdoba, Argentina. In said study 22 out of 34 neighborhoods were surveyed randomly. Subjects ≥18 years old, both sexes who went voluntarily to the ambulatory attention on the day and time previously told on different media channels, were included. Participants who were previously diagnosed with psychiatric diseases, dementia and/or depression (based on Diagnostic and Statistical Manual of Mental Disorders, 4th edition criteria), and stroke as well as those who were not able to complete the required protocol tests since they had sensory, motor, or neurological disorders—that stopped these participants from doing the tests—were excluded. The sample was formed by 1460 participants voluntarily called. A closed-answers questionnaire was used (national survey of the risk factors for nontransmissible diseases) [5] in which we asked demographic questions (sex, age), family background, socioeconomic situation, physical activity frequency, and RF (HTN, dyslipemia, obesity, and diabetes). The participants were divided into 3 different groups according to their schooling level: Level 1 (primary school finished/unfinished), Level 2 (secondary school finished/unfinished), and Level 3 (tertiary school/college).

### 2.2. Definitions and Cut-Off Values

The variables we took into account were (1) blood pressure (diastolic and systolic) taking as an average value the last 2 out of 3 measurements of a seated subject using a validated digital sphygmomanometer with size cuff “ComFit” adjustable according to the arm circumference (OMRON HEM-714 INT); (2) anthropometric measurement was standardized: body weight (recorded to the nearest 0.1 kg), body height (m), and waist circumference (cm) measuring the midpoint between the lowest rib and the iliac crest in which the participants were standing with non-forced exhalation. In all cases the same weighting machine (Co.Ar.Mc. N.R. model E 1001 with range from 5 to 150 kg) and tape measure were used. Body mass index (BMI) was calculated according to Quetelet formula. Blood pressure and anthropometric values were measured without knowledge of the cognitive score and vice versa; And (3) the glucose, triglyceride, and lipoproteins concentrations were measured using enzymatic standard techniques in venous blood. Blood pressure was recorded according to national and international guidelines and HTN was defined according to the guidelines of the Argentine Society of Cardiology [6] consistent with the European guidelines (European Society of Hypertension and European Society of Cardiology-ESH/ESC) [7]. All the patients that were diagnosed with HTN were on antihypertensive drugs, not so the diabetic and dislipemic patients. For the waist circumference value in metabolic syndrome, abdominal obesity was defined according to the criteria and the cut-off points of the American Heart Association (AHA) and the National Heart, Lung and Blood Institute 2005 (NHLBI) [8]. For the diabetes diagnoses, the American Diabetes Association criteria were used [9]. For the venous blood sample, the participant did a >10-hour fasting so as to obtain samples of glycemia, total cholesterol (TC), HDL-cholesterol, LDL-cholesterol (measured but not calculated by Friedewald's formula), and triglycerides (TG). The cut-off points for a dyslipemia diagnosis were TC ≥ 200 mg/dL, HDL ≤ 50 mg/dL in women and ≤40 mg/dL in men and TG ≥ 150 mg/dL.

### 2.3. Neuropsychological Assessment

Anxious or depressive symptoms were assessed using the 14-item anxiety-depression scale [10]. The item score was added individually and together with a maximum score to 42 points, considering the presence of symptoms when the results was >11 points. Aquestionnaire was administered by an interviewer in order to identify any behavior that biases the results. All subjects underwent testing for cognitive function using the Minimal Cognitive Examination (MCE) (described in previous studies) [11]. The MCE includes tasks measuring attention, orientation, short- and long-term memory, semantic memory, praxis, language, visual construction, and executive function. Designed for a quick and easy approach to the cognitive state, the MCE is composite of the 5 tests: (a) minimental statement examination (MMSE) [12] in which we followed the recommendations of the Neuropsychological Working Group of the Argentine Society of Neurology [13] (no impairment ≥27 points; cognitive impairment 25 to 26 points and potential dementia ≤24 points); (b) Benton orientation questionnaire; (c) Clock Drawing Test; (d) alternating series test; (e) abbreviated form of the Boston Naming Test [14]. These tests were grouped according to the area of study (global cognition: MMSE and Benton Orientation Questionnaire; executive function: clock drawing test and alternating series test; memory: Boston naming test).

### 2.4. Ethical Considerations

Field work was done in the medical centers of each neighborhood. All interviewers were trained before the study began. The tests were blindlyexamined by two neuropsychologists.

The participants signed an informed consent before participating in the trial. The trial protocol and the informed consent were approved by an independent ethic committee. The trial was done pursuant to the GCP (good clinical Practice), the local regulations, and the Declaration of Helsinki and its amendments.

### 2.5. Design and Statistical Analyses

This is an epidemiological, cross-sectional, and observational study. While the relative frequencies of the categorical variables were expressed in percentages, the continuous variables were expressed with mean ± standard deviation (SD). For paired samples, an analysis using two variables per category using the *χ* ^2^ test or ANOVA was used. Odds ratio (95% confidence intervals (CIs)) was used as a measure of risk. It depended on the nature of the pair sample compared: they were adjusted by age, sex, and schooling level. In all cases, the trust level was 95%.

## 3. Results

In this study 1365, ≥18-year-old participants from both sexes were included.  Table 1 shows the characteristics of the sample (age, schooling level, anthropometry, blood pressure, and blood chemistry) separated by sex. The value of the systolic blood pressure (SBP) was higher in men than in women (men 142.63 ± 20.9 versus women 132.7 ± 22.5; *P* < 0.01). The value of the plasmatic TG level was also higher in men than in women (180.3 ± 12.7 versus 135.2 ± 85.2; *P* < 0.01). From the total cohort 24.5% were ≥60 years old and 9.2% ≥70 years old.

Table 2 shows the prevalence of RF related to the cognitive results and the anxiety/depression scale.

**Figure 1:**
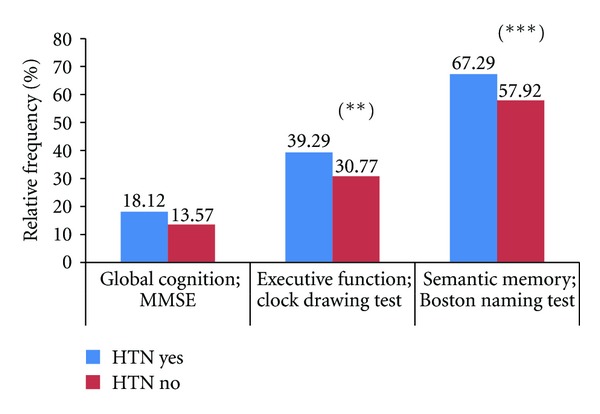
Cognitive tests relative frequencies between hypertensive and non-hypertensive participants. HTN; hypertension, (∗) *P* < 0,02; (∗∗) *P* < 0,03.

**Figure 2:**
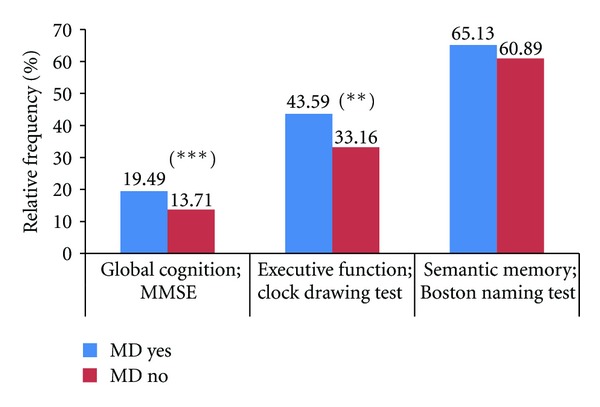
Cognitive tests relative frequencies between Diabetes and non-diabetes participants. MD; mellitus diabetes, (∗) *P* < 0,02; (∗∗) *P* < 0,03.

Table 3 shows the results of the cognitive tests in which anxiety disorders were higher in women than in men (score 7.17 versus 4.5; *P* < 0.01). Moreover, 14.5% showed MMSE ≤ 24, 34.6% (*n* = 473) showed executive failures and 45.8% (*n* = 626) showed memory failures. Among the RF, HTN was related with the cognitive domains: executive function (39.29% versus 30.77%; *P* < 0.03) and memory (67.29% versus 57.92%; *P* < 0.02) (Figure 1); diabetes was related to global cognition (19.49% versus 13.71; *P* < 0.03) and executive function (43.59% versus 33.16%; *P* < 0.004) (Figure 2); obesity was related only with memory. Dyslipemia did not show any relationship with any cognitive domain. Clock drawing test and Boston naming test identified more cognitive disorders than the MMSE alone (clock drawing test versus MMSE OR 2.23; IC95% 2.14–4.00; *P* < 0.0001 and Boston naming test versus MMSE OR 9.30; IC95% 6.76–12.79; *P* < 0.0001). When the trial was adjusted by age, sex, and schooling level, the ≥70-year-old group was the only one that was different from the other groups and showed a higher cognitive impairment (35.1%, *P* < 0.0001).

## 4. Discussion

As long as life expectancy increases, there will also be a correlative increase in cognitive impairment and dementia. This fact makes age the main RF for dementia. That is why, in most of the previous studies about this topic, the cohorts are 60–70 years old or older. However, some trials showed that patients may have cognitive impairment before that age (approximately 40 years old). Singh-Manoux et al. [4] did a 10-year followup over a cohort of 7000 participants between 45 and 49 years old and found that there was a −3.6% decrease in the cognitive skills (especially executive function and short-term memory) compared to the younger groups. Salthouse et al. [15] found that healthy adults (20–30 years old) with appropriate schooling level may have some aspects of cognitive decline. The analysis of our sample (18–88-year-old participants) showed that 14.5% scored ≤24 pts in the MMSE which was compatible to a possible cognitive impairment or dementia. When the other MCE tests, which are more specific to study executive functions and memory, were used, said percentage increased to 45.6%. These high percentages—which may be explained partially by low schooling level of the sample—were controlled by age, sex, and depression. These variables are confusing when relating predictor variables with cognitive function. In the adjusted model, only the ≥70-year-old group was different (35.1%, *P* < 0.0001).

Together with these data, HTN, diabetes, and obesity, among others that were not considered in this study, may add to age a negative impact over cognition. In a 20 years followup, Elias et al. [16] showed that young adults are as sensitive to high blood pressure related cognitive decline as elderly adults. These variables are inversely related to cognitive performance and it affects mainly the executive domains dependant on the prefrontal cortex [17, 18]. This is a secondary damage causing the disconnection of the sub-corticocortical circuits done by hipoperfusion of the brain white matter. Since 1970 there have been more than 20 longitudinal trials that found a causal relationship between the RF and cognitive impairment, vascular dementia, and AD [19].

From the four studied RF (HTN, dyslipemia, diabetes, and abdominal obesity), the only one that was not related to cognitive impairment was dyslipemia. Even though this had a significant relationship using the three tests separately, in the grouped test on executive functions and semantic memory, a higher number of participants with impairment were found. The importance of using the MCE (which includes the MMSE, clock drawing test, Boston naming test, etc.) is to create a wider field for the cognitive cortical disorders, (e.g., Alzheimer's type dementia). Clock drawing test (OR 2.23) and Boston naming test (OR 9.3) had a significant increase in identifying probable neurodegenerative cortical dementia compared to the MMSE (*P* < 0.0001) alone. These tests work on the visual constructive alterations—an early impairment in AD—and semantic memory—kept in the normal aging and subcortical dementias. Thus, the strict control of the RF is not only important for the vascular dementia prevention, but also Alzheimer's type dementia [20, 21]. In many brain autopsy reports, AD was found in 60–90% of the cases [22, 23]. In a previous trial in which 202 hypertensive patients were studied, dementia was diagnosed in 8.9% (50% of the patients that according to the MCE were prone to dementia) in which 72% of those patients had Alzheimer's type dementia [24]. Likewise, longitudinal trials found that there was a higher prevalence of Alzheimer's type dementia in patients with HTN or grouped RF [25–27].

From these new findings, we have to modify and improve future studies design using another model that let us clarify the covariability of the RF more precisely in cognitive test results. In this way, this can be a considered a limitation in our study. 

In this way, RF control not only prevents heart and brain damages, but also cognitive disorders and especially dementia. Even though there are drugs that evidence their control over dementia symptoms (cognitive and/or behavior), the most accepted preventive strategy seems to be vascular RF control. If we could delay the beginning of dementia by 5 years, the cases of dementia would decrease in 1 million in 10 years [28]. The results showed that we need to (a) “expand” the early identification of the RF and the cognitive impairments to all age groups; (b) “highlight” the importance of using other tests than the MMSE to improve disorder identifications; (c) “assume” that controlling RF, and especially HTN, helps to prevent new cases of dementia especially Alzheimer's type dementia cases. These have a great impact on public health and force to consider more aggressive health policies. There is scientific technological progress that helps us solve acute vascular problems and helps to increase life expectancy, but RFs have not been controlled yet. This last one and the early identification of cognitive impairment are our future challenges.

## Figures and Tables

**Table 1:** General characteristics of the participants in each of the studied variables and difference between sexes (values in mean ± SD).

Variables	Totals	Men	Women	*P* value
Sample (*n*)	1365	332	1033	
Age (years)	49 ± 15,6	48,6 ± 16,2	47,1 ± 15,7	
Sex		24,3%	75,7%	
Schooling level (%)				
Level 1	50,4	51,8	49,9	ns
Level 2	35,7	35,2	35,3	ns
Level 3	14,3	12,9	14,8	ns
Anthropometry				
Weight (kg)	75,9 ± 17,9	83,7 ± 16,5	73,4 ± 17,6	ns
WC (cm)	96 ± 15	99,3 ± 13,4	94,9 ± 15,3	ns
BMI (kg/m^2^)	29,4 ± 6,5	28,7 ± 5,1	29,6 ± 6,9	ns
BP (mm Hg)				
SBP	135,2 ± 22,5	142,6 ± 20,9	132,7 ± 22,5	<0,01
DBP	80 ± 12,5	83,1 ± 12,6	78,9 ± 12,3	ns
Blood chemistry (mg/dL)				
Glycemia	90,2 ± 29,4	98,3 ± 37,3	90,2 ± 26,1	ns
Total cholesterol	196,2 ± 44,1	198,8 ± 46,2	195,2 ± 43,4	ns
HDL	49,9 ± 11,8	45,4 ± 0.9	51,2 ± 12	ns
LDL	124,4 ± 35,2	127,5 ± 36,9	123,4 ± 24,7	ns
Triglycerides	146,1 ± 96,8	180,3 ± 12,7	135,2 ± 85,2	<0,01

Level 1: (primary school finished/unfinished), Level 2: (secondary school finished/unfinished), and Level 3: (tertiary school/college); WC: waist circumference; BMI: body mass index; BP: blood pressure; SBP: systolic blood pressure; DBP: diastolic blood pressure; HDL: high density lipoprotein; LDL: low density lipoprotein.

**Table 2:** Prevalence of the RF related to the cognitive test results.

Cognitive test	Hypertension		Diabetes		Obesity		Dislipemia	
	Yes	No	Yes	No	Yes	No	Yes	No
Anxiety plus depression	7,78 (33)	10,86 (24)	11,28**(22)	6,87 (80)	8,43 (71)	6,14 (32)	7,44 (32)	7,49 (69)
MMSE	18,12 (77)	13,57 (30)	19,49**(38)	13,71 (160)	15,18 (128)	13,41 (70)	15,81 (68)	13,98 (129)
Clock drawing	39,29** (167)	30,77 (68)	43,59*** (85)	33,16 (387)	36,54 (308)	31,61 (165)	37,67 (162)	33,48 (309)
Boston naming	67,29* (286)	57,92 (128)	65,13 (127)	60,89 (710)	63,82** (538)	57,97 (302)	62,56 (269)	61,28 (565)

The values are percentages and absolute pathological findings frequencies.

*P < 0,02; **P < 0,03; ***P < 0,004.

**Table 3:** Results of the anxiety/depression scale and cognitive tests and differences between sexes (values in mean ± SD of the score of each test).

Variables	Totals	Men	Women	*P* value
Behaviour				
Anxiety	6,52 ± 4,5	4,4 ± 3,7	7,1 ± 4,6	<0,01
Depression	4,37 ± 4,5	3,1 ± 3,3	4,7 ± 4,7	ns
Cognition				
Global cognition				
Minimental test	27,3 ± 3,2	26,9 ± 3,4	27,4 ± 3,1	ns
Benton's test	20,7 ± 3,0	20,4 ± 3,3	20,8 ± 2,9	ns
Executive function				
Clock drawing test	5,5 ± 1,8	5,7 ± 1,6	5,5 ± 1,8	ns
Alternating series test	1,6 ± 0,59	1,6 ± 0,6	1,6 ± 0,5	ns
Memory				
Boston naming test	8,22 ± 2,7	8,3 ± 2,9	8,1 ± 2,7	ns
